# Assessment of the midgut microbiota in foragers of the stingless bee *Melipona scutellaris* following short-term sublethal exposure to imidacloprid

**DOI:** 10.1371/journal.pone.0339982

**Published:** 2025-12-29

**Authors:** Patricia Azevedo, Lucas Miotelo, Manuela Oliveira Ramalho, Tatiane Caroline Grella, Osmar Malaspina, Matthew Hudson, Roberta Cornélio Ferreira Nocelli, Maria Imaculada Zucchi

**Affiliations:** 1 Universidade Estadual de Campinas (UNICAMP), Instituto de Biologia, Grupo de Genética e Genômica da Conservação, Programa de Pós-graduação em genética e biologia molecular, Piracicaba, São Paulo, Brasil; 2 Universidade Estadual Paulista (UNESP), Instituto de Biociências, Departamento de Biologia Geral e Aplicada, Programa de pós-graduação em Ciências Biológicas: Biologia Celular, Molecular e Microbiologia, Rio Claro, São Paulo, Brasil; 3 West Chester University, Department of Biology, West Chester, Pennsylvania, United States of America; 4 University of Illinois, Department of Crop Sciences, Urbana, Illinois, United States of America; 5 Universidade Federal de São Carlos (UFSCar), Departamento de Ciências da Natureza, Matemática e Educação, Programa de Pós-graduação em agricultura e ambiente, Araras, São Paulo, Brasil; 6 Agência Paulista de Tecnologia do Agronegócio (APTA) – Polo Centro Sul, Piracicaba, São Paulo, Brasil; Institute of Apicultural Research, CHINA

## Abstract

The insecticide imidacloprid, widely used in Brazil, is known to have adverse effects on bee populations at both lethal and sublethal doses and concentrations. While previous studies have reported disruption of bee gut microbial communities following pesticide exposure, the effects of short-term sublethal exposure in stingless bees remain unclear. This study evaluated the impact of sublethal concentrations of imidacloprid (LC_50/100_ and LC_50/1000_) on the midgut microbiota composition of the stingless bee *Melipona scutellaris*. Using metabarcoding of the V3 and V4 regions of 16S rRNA to characterize bacterial community composition and diversity, we analysed midgut bacterial communities of bees collected directly from hives (T0 group) and after 24 and 48 hours of oral imidacloprid exposure. Our results showed that, after 24 and 48 hours of exposure, there were no significant changes in midgut bacterial diversity or composition between bees exposed to sublethal concentrations of imidacloprid and their respective controls. In contrast, we observed clear differences in both diversity and abundance between the T0 group and control groups, especially after 24 hours of incubation. These differences were primarily associated with shifts in *Acetobacteraceae* family, likely reflecting the influence of the artificial sucrose diet provided under laboratory conditions. It is important to note that our findings pertain only to acute effects. Future studies would benefit from extending the duration of exposure and including a broader range of toxicological endpoints to build on these findings. Our results also underscore the importance of evaluating both exposure period and dietary conditions when investigating pesticide impacts on bee microbiota.

## 1. Introduction

Due to its climatic and environmental characteristics and the presence of several biomes within its territory, Brazil has some of the greatest megadiversity in the world [1]. This diversity extends to bee species, with approximately one-quarter of the estimated 20,000 species worldwide being native to the country [2,3]. However, bee populations are declining due to several factors, including land use changes, disease outbreaks, climate change, and pesticide use [4,5].

Imidacloprid, a neonicotinoid class of synthetic insecticides, is widely used in Brazilian agricultural crops, including citrus, sunflower, and the *Solanaceae* plant family [6]. Its mechanism of action involves mimicking acetylcholine and acting on nicotinic acetylcholine receptors (nAChRs), prolonging their activation and causing hyperexcitation, paralysis, and insect death [7]. As a systemic insecticide, it is transported through the plant’s vascular system and can be present in exudates and guttation, which bees use as a source of food and water [8,9]. Additionally, its high-water solubility and persistence can result in the product remaining in soil and groundwater for months to years [10].

Although bees are not the intended target, they have been severely affected by neonicotinoids worldwide [11]. The decline in bee population linked to neonicotinoid use has led to a ban on the use of the insecticide in open-field crops in Europe and some areas of the United States, the Philippines, and Canada [12]. In Brazil, neonicotinoids remain widely used, with imidacloprid ranking ninth in total sales in 2020, with 9,401.65 metric tons of active ingredient sold [13]. Several studies have shown that the use of imidacloprid affects bees’ health, causing foraging difficulties, neurological impairment, immunological suppression, and metabolic reactions, at both lethal and sublethal doses and concentrations [14–18]. This issue is particularly relevant for non-*Apis* bees, whose responses to pesticides can differ from those of the model organism *Apis mellifera*, yet they are often not adequately considered in risk assessment processes [19,20].

To enhance protective measures for bees, the Brazilian Institute of Environment and Renewable Resources (IBAMA) started reevaluating pesticide-active ingredients suspended in the European Union in 2012. Moreover, the institute organized a working group that developed an environmental risk assessment manual for bees [21], which identified the stingless bee *Melipona scutellaris* as one of the priority species to be tested for inclusion in pesticide risk assessment tests. Notably, *M. scutellaris* possesses a significant morphological adaptation for pollination, known as buzz pollination, which enhances its potential for large-scale pollination [22]. Furthermore, *M. scutellaris* is listed on the Red List of endangered species by Chico Mendes Institute for Biodiversity Conservation (ICMBio) due to its decline in both population numbers and original distribution. This underscores the importance of conducting studies with this species [23].

In general, insects possess several defense mechanisms against pathogens and harmful external agents, such as pesticides. These include the digestive system, which uses the intestinal epithelium as a primary barrier due to its specialized cells [24], the peritrophic membrane, which acts as a physical barrier against parasites and bacteria [25], and the gut microbiome, which contains about one billion bacteria [26]. Together, they promote the degradation of toxic compounds, defense against pathogens and parasites, behavioral regulation, development, and host immunity [27–30].

Studies show that bee gut microbial activity plays an important role in modulating the immune system, inducing antimicrobial peptide gene expression [31], protection against viruses [32], mites and parasites like *Varroa destructor* [33], and *Nosema ceranae* [34,35]. However, the literature indicates that pesticides can compromise the microbial community of *Apis mellifera* [36–40]. Research on potential alterations in the microbiota of stingless bees due to pesticide exposure is still limited. To date, only two studies have examined this issue in *Partamona helleri* [41,42]. Nonetheless, this remains a significant knowledge gap, particularly with respect to sublethal pesticide effects on stingless bees. Based on this scenario, the present study evaluated the effects of two sublethal concentrations of imidacloprid on the midgut microbiota of the stingless bee *M. scutellaris* over 24 and 48-hour exposures. This marks the first investigation into the bacterial community of *Melipona scutellaris* under exposure to sublethal concentrations of pesticides.

## 2. Materials and methods

### 2.1. Biological material

Forager bees of *M. scutellaris* were collected at the entrance of three non-parental colonies in the meliponary (22° 23′ 49″ S and 47° 32′ 37″ W) at São Paulo State University (UNESP), Institute of Biosciences, Rio Claro, SP, Brazil. The toxicity bioassays were performed following the guidelines for testing chemicals in bees [43], with adaptations for stingless bees relating to temperature, feeder type, humidity, and the number of bees collected, as previously described by Miotelo et al. [44].

The bees were collected in plastic cages (250 mL, 9 cm × 7 cm) previously drilled on the sides for proper air circulation, and with a hole in the lid to insert a 2 mL microtube, also previously drilled on the side. The microtubes, called feeders, were filled with sucrose solution (water and sugar: 50% w:v) to provide food for the collected bees.

### 2.2. Toxicity bioassays

Ecotoxicological bioassays were conducted via oral exposure using PESTANAL^®^ imidacloprid (C_9_H_10_CIN_5_O_2_, purity ≥ 98.1%, Sigma-Aldrich) as the analytical standard. A stock solution of 1000 ng/μL was prepared by dissolving 10 mg of imidacloprid in 10 mL of a solvent mixture containing 85% water and 15% acetone. Sublethal concentrations were selected based on the 24-hour LC_50_ of 2.01 ng a.i./μL previously determined for foragers of the stingless bee species *M. scutellaris* [45]. Two test concentrations, LC_50/100_ (0.0201 ng a.i./µL) and LC_50/1000_ (0.00201 ng a.i./µL), were obtained by serial dilution of the stock solution using a 50% (w:v) sucrose solution, which was also used to feed the control group. These concentrations reflect environmentally relevant levels, consistent with imidacloprid residues detected in nectar (0.015–0.0076 ng/µL) [46]. Additionally, in alignment with OECD guidelines, we selected 24- and 48-hour exposure periods, which are widely adopted in standardized toxicity testing. As highlighted by Fischer [47], innovative strategies are essential to advance current pollinator risk assessments. In this context, the integration of microbiome analysis into acute exposure protocols provides valuable insights into early microbial responses, potentially serving as sensitive biomarkers of chemical exposure.

**Fig 1:**
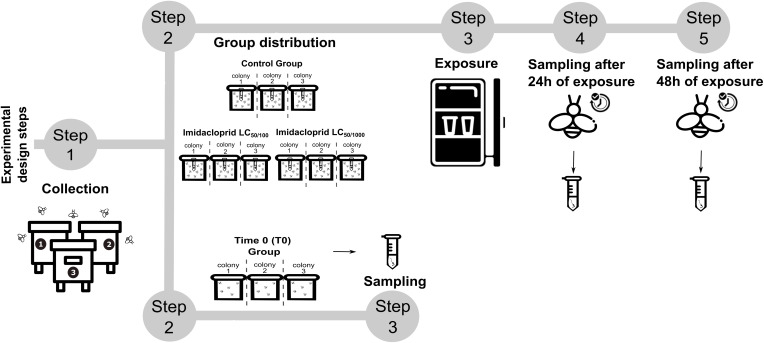
Experimental design. The exposure timeline is divided into the following steps: **(1)** Bees were collected in plastic cages; **(2)** They were assigned to experimental groups as follows: foraging stingless bees were orally exposed to two imidacloprid concentrations (LC_50/100_ = 0.0201 ng a.i./µL and LC_50/1000_ = 0.00201 ng a.i./µL), the control group, and the T0 group. Only the groups exposed to imidacloprid received a sucrose solution containing their respective concentrations throughout the entire assay. The control group received an uncontaminated sucrose solution, while bees in the T0 group were not exposed to any laboratory conditions. **(3)** Bees were placed in an incubator, including both the sublethal concentration and control groups, and were then subjected to midgut dissection at **(4)** 24 and **(5)** 48 hours post-insecticide exposure. Additionally, **(2)** nine bees were set aside as the Time 0 (T0) group, and **(3)** their midguts were sampled without exposure to laboratory conditions.

Ninety-nine forager bees were collected and divided into experimental groups: thirty bees per treatment, arranged as three replicates of ten bees each, with each replicate originating from a different colony. Additionally, three foragers for each colony (nine bees total) were immediately dissected to obtain midguts for baseline microbiome validation (Time 0 group). Bees were housed in cages of ten individuals and maintained in a B.O.D. incubator at 28°C ± 1°C and 70% ± 5% relative humidity. Food was provided *ad libitum*, with the control group receiving an uncontaminated sucrose solution. At each time point, 24- and 48-hour, nine bees per group (three for each colony) were dissected and had their guts stored at −80°C until the DNA extraction. During dissections, sterilized materials were used, including autoclaved Petri dishes, tweezers, and scissors. In total, 63 midguts were collected: nine from the Time 0 group, and 27 each from the three experimental groups (control, LC_50/100_, and LC_50/1000_) across the two exposure times (Fig 1).

### 2.3. DNA extraction, library construction, and sequencing

Bees’ midgut DNA was extracted according to the CTAB method [48] as described by Moro et al. [49], and the integrity of the extracted DNA was assessed by electrophoresis on 1% agarose gel. The DNA was then sent to the Genomics Facility Center (ESALQ, Piracicaba, SP, Brazil) for library construction and 16S rRNA amplicon sequencing according to the Illumina preparation guide [50]. A two-step PCR was used to construct the libraries. The V3 and V4 regions of the 16S rRNA gene were amplified with 25 cycles using the primers 341F (5’-CCTACGGGNGGWGCAG-3’) and 805R (5’-GACTACHVGGGTATCTAATCC-3’) with adapters, as recommended in the Illumina 16S Metagenomic Sequencing Library Preparation protocol [50]. These regions were chosen because they provide a good balance of taxonomic resolution and broad bacterial detection, enabling accurate and comprehensive profiling of the bee gut microbiota [51,52]. The PCR products were then purified using magnetic beads (Agencourt AMPure XP^®^ - Beckman Coulter) and, after purification, a second PCR was performed to anneal the adapters using the Nextera Index Kit^®^. Finally, the PCR products were purified again, quantified on Nanodrop, and normalized to 50 ng/µL. Three negative controls were also included in the library preparation, which were sequenced alongside the actual samples. These controls were added to identify and later eliminate any contaminants that may have originated during the data generation process. The pool was diluted to a final concentration of 2 nM, prepared according to Illumina protocol, and sequenced using Illumina Miseq^®^ 2 × 300 bp (v3 600 cycles). In total, 63 samples and three negative controls were sequenced and subjected to bioinformatics analysis.

### 2.4. Bioinformatic analysis

The demultiplexed sequencing was subject to primary quality control using FastQC software (version 0.11.9), and the analysis showed good quality statistics for all samples. Raw reads are available in the NCBI Sequence Read Archive under BioProject PRJNA1020169, BioSamples SAMN37516683–SAMN37516747, and SRA accessions SRR26149385–SRR26149434. After, the data were analysed using *Qiime2/2022.8* [53]. Quality control and feature table construction were performed using the Dada2 plugin, with both forward and reverse 16S rRNA reads entered the pipeline. First, denoising was done with the truncation by read length parameters for quality filtering as follows: *--p-trim-left-r 6/ --p-trim-left-f 6/ --p-trim-len-f 284/ --p-trim-len-r 253*. The taxonomic classification of the features was then predicted using the q2-feature-classifier extract-reads plugin. The sequenced target sequences were extracted as follows: *--p-f-primer CCTACGGGNGGCWGCAG/ --p-r-primer GACTACHVGGGTATCTAATCC/ --p-trunc-len 282*. The Naive Bayes classifier was then trained and tested using SILVA version 132 [54]. Subsequently, to evaluate the purity of the reagents and manipulation during the experiment, the *Decontam* package [55] from R software [56] was used to remove contaminant sequences provided by the three negative controls sequenced that passed through all steps of DNA extraction. Next, all the mitochondrial and chloroplast sequence reads were removed from the feature tables. Finally, the phylogenetic tree was performed using the fragment-insertion SEPP [57].

### 2.5. Statistical analysis

Alpha and beta diversity were calculated using *Qiime2/2022.8* [53]. Alpha diversity, which assesses richness and diversity within a sample, was measure with a maximum depth of 1000 (*--p-max-depht 1000*) by calculating the Shannon entropy (quantitative community richness), Evenness (species relative abundance), the Chao1 (species richness focusing on rare and unique taxa), the Simpson index (richness and relative abundance of species), and the number of ASVs (Amplicon Sequence Variants) observed. Shannon and evenness alpha diversity metrics were analysed using the Wilcoxon Signed-Rank Test (Wilcoxon SRT) for pairwise comparisons in *Qiime2* [53], while Chao1 and Simpson indexes were calculated in R [56] and similarly compared pairwise with the Wilcoxon SRT.

Beta diversity, which compares microbial composition between samples/groups, was assessed using Bray-Curtis (quantitative community dissimilarity based on abundance), weighted UniFrac (quantitative dissimilarity incorporating phylogeny and abundance), and unweighted UniFrac (qualitative dissimilarity incorporating phylogeny based on presence/absence) metrics. The beta diversity metrics were analysed using PERMANOVA for multivariate community data. In addition, the SIMPER (SIMilarity of PERcentages) analysis in PAST3 software [58] was used to identify the ASVs responsible for the between-group differences. This analysis calculates the contribution of each ASV species (%) between two groups through the Bray-Curtis dissimilarity matrix. ANCOM (Analysis of Composition of Microbiomes) analysis was implemented as a plugin in *Qiime2* [59] and used to identify differentially abundant taxa between groups that presented differences in alpha and beta diversity. The rarefaction curve and the relative frequency of the top 10 genera ranked by relative abundance were calculated with the R software [56]. The Figs were generated using R software [56].

## 3. Results

**Fig 2:**
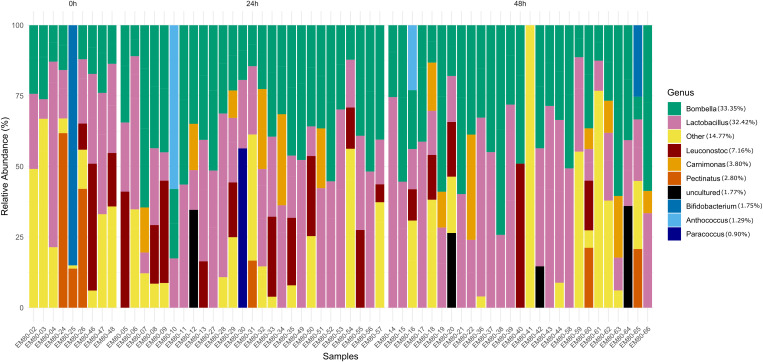
Composition of the midgut microbiota across experimental groups. The bar chart illustrates the relative abundance of predominant bacterial ASVs in the midgut microbiota across all experimental groups. Each vertical bar represents the microbial community composition of an individual sample, with distinct colors denoting different bacterial genera. The legend indicates the overall relative frequency of the top 10 genera, ranked by relative abundance, across all analysed samples.

The structure and composition of the midgut microbiota of *M. scutellaris* exposed to sublethal concentrations of the insecticide imidacloprid, and the validation between the control group and T0 group, were evaluated by 16S rRNA amplicon sequencing. A total of 6,020,450 reads were obtained from the 63 samples and the three negative controls, including both forward and reverse reads. After filtering out low-quality sequences, negative controls, mitochondrial and chloroplasts, 146,408 reads remained, from which 422 Amplicon Sequence Variants (ASV) were recovered. Alpha and beta diversity analyses were first performed on all samples to verify the data structure (S1 File - PCoA analysis) and the most abundant ASVs across all groups (Fig 2). The midgut microbiota was primarily dominated by two core bacterial genera: *Bombella* (33.35%) and *Lactobacillus* (32.42%), both of which were consistently present across all samples. *Leuconostoc* (7.16%) emerged as the third most prevalent genus detected in nearly all samples. These findings indicate the presence of dominant bacterial genera in the midgut, with *Bombella* and *Lactobacillus* playing particularly significant roles in the microbial community structure.

### 3.1. Midgut microbiota composition comparing the sublethal concentrations of imidacloprid and the control groups

**Fig 3:**
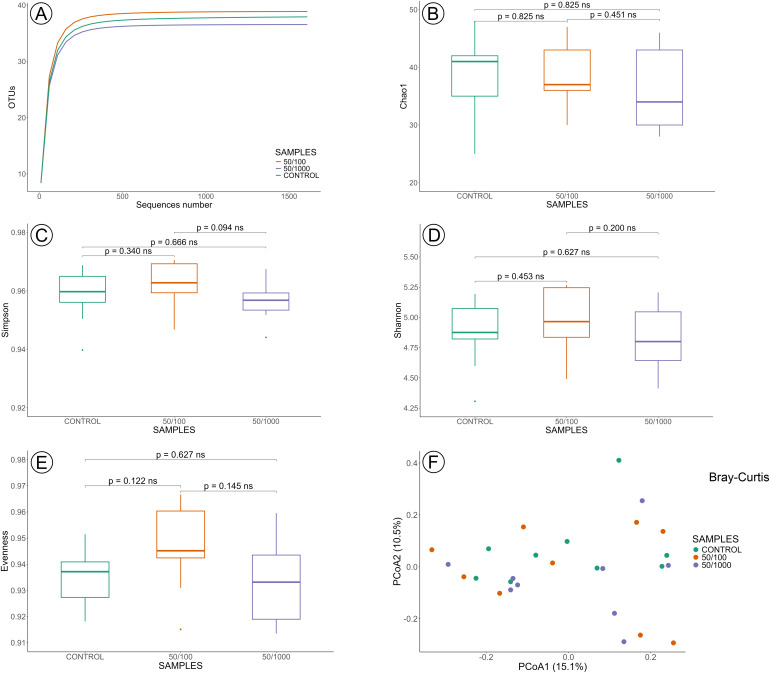
Gut microbiota diversity at sublethal concentrations of imidacloprid (LC_50/100_ and LC_50/1000_) after 24 hours of exposure. **(A)** Rarefaction of samples. Alpha diversity was evaluated based on the Chao1 **(B)**, Simpson **(C)**, Shannon **(D)**, and Evenness **(E)** indices of OUT levels. PCoA analysis of beta diversity was based on the Bray-Curtis results **(F)**. Statistical significance between groups was analysed by the Wilcoxon SRT test (p > 0.05). ns = not significant.

**Fig 4:**
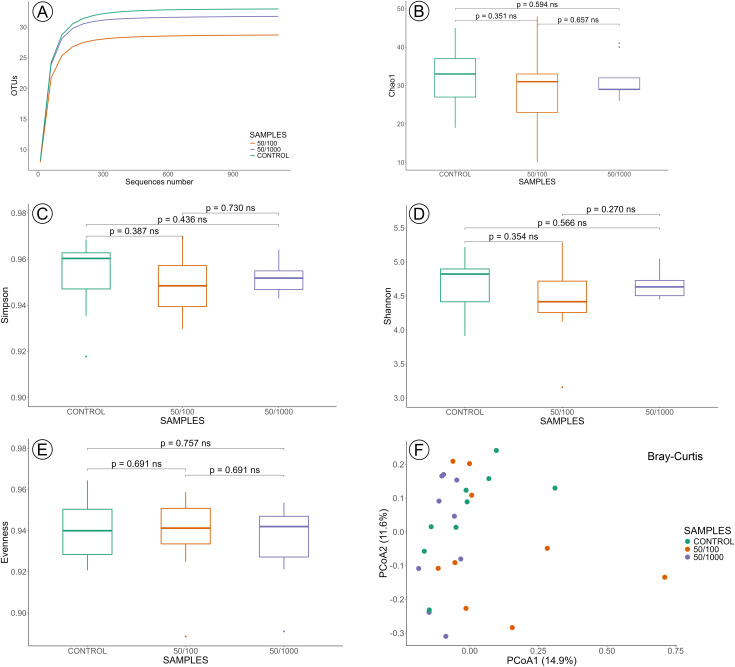
Gut microbiota diversity at sublethal concentrations of imidacloprid (LC_50/100_ and LC_50/1000_) after 48 hours of exposure. **(A)** Rarefaction of samples. Alpha diversity was evaluated based on the Chao1 **(B)**, Simpson **(C)**, Shannon **(D)**, and Evenness **(E)** indices of OUT levels. PCoA analysis of beta diversity was based on the Bray-Curtis results **(F)**. Statistical significance between groups was analysed by the Wilcoxon SRT test (p > 0.05). ns = not significant.

The analysis comparing the sublethal concentrations and the control groups at 24 and 48 hours was performed on 54 samples: control 24 h = 9, control 48 h = 9, LC_50/100_ 24 h = 9, LC_50/100_ 48 h = 9, LC_50/1000_ 24 h = 9 and LC_50/1000_ 48 h = 9. The rarefaction curves for all groups reached a plateau, indicating that the sequence data adequately represent the taxa diversity present in the samples analysed in this section, validating the sequencing depth downstream diversity analysis (Fig 3A and Fig 4A).

Comparisons of alpha diversity across sublethal concentrations of imidacloprid at 24- and 48-hour showed no statistically significant differences in the Chao1 (Fig 3B and Fig 4B), Simpson (Fig 3C and 4C), Shannon (Fig 3D and 4D), and Evenness (Fig 3E and 4E) indexes (S2 File – Alpha indexes). This convergence across multiple diversity metrics indicates that both the estimated species richness (Chao1), the balance of abundance among taxa (Simpson and Shannon), and the evenness of the community structure were similar between groups. These results suggest that the imidacloprid did not affect the internal diversity nor distribution of taxa within the communities. Beta diversity analysis also showed no influence of the sublethal concentrations of imidacloprid in the bacterial community according to Bray-Curtis (Fig 3F and Fig 4F), weighted Unifrac (24h: pseudo-F = 0.385, p-value = 0.939; 48h: pseudo-F = 1.270, p-value = 0.218), and unweighted Unifrac metrics (24h: pseudo-F = 0.726, p-value = 0.833; 48h: pseudo-F = 0.902, p-value = 0.559), at all times of exposure, when compared to control groups. These findings suggest that the tested variable did not significantly alter the microbial community structure or phylogenetic diversity between groups.

### 3.2. Midgut microbiota composition comparing the T0 group and control groups at 24 and 48 hours

**Fig 5:**
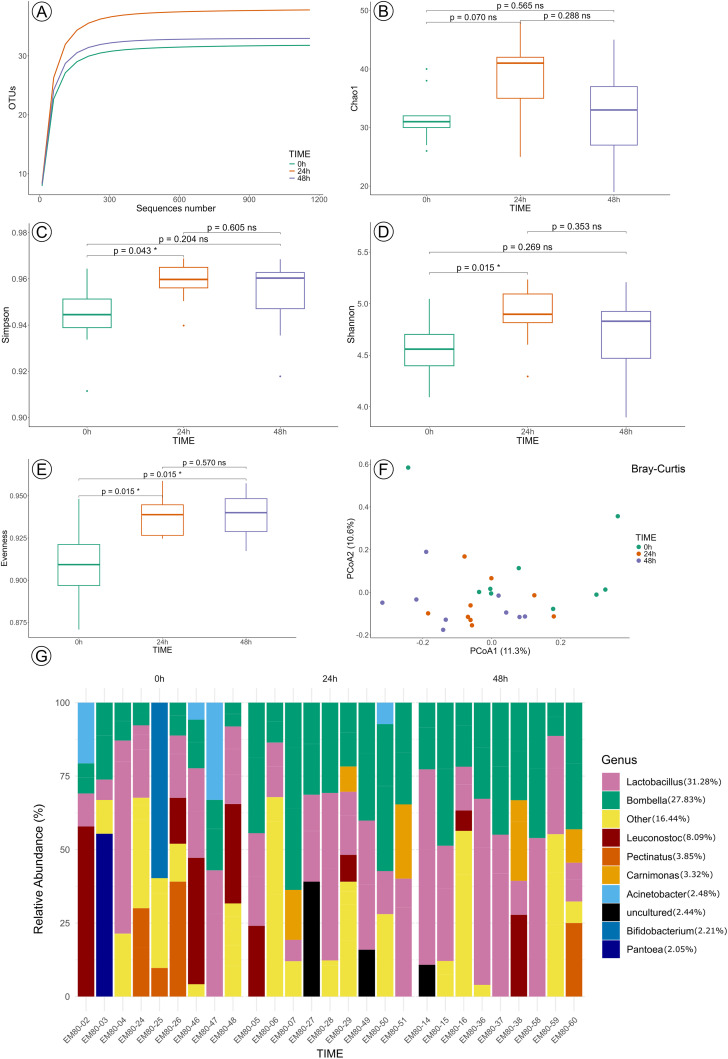
Gut microbiota diversity of T0 and control groups after 24 and 48 hours of exposure. **(A)** Rarefaction of samples. Alpha diversity was evaluated based on the Chao1 **(B)**, Simpson **(C)**, Shannon **(D)**, and Evenness **(E)** indices of OUT levels. PCoA analysis of beta diversity was based on the Bray-Curtis results **(F)**. Statistical significance between groups was analysed by the Wilcoxon SRT test (p > 0.05). ns = not significant. Microbiota composition showing the relative abundance of predominant bacterial ASVs **(G)**. Each vertical bar represents an individual sample’s microbial community, with colors indicating different bacterial genera. The legend displays the overall relative frequency of each genus across all samples.

The analysis comparing the T0 group and control groups at 24 and 48 hours was performed on 27 samples: T0 = 9, control 24 h = 9, and control 48 h = 9. The midgut microbiota composition for the T0 group and control at 24 and 48 hours included the genus *Lactobacillus* (31.28%) and *Bombella* (27.83%) as the main bacterial members in all samples (Fig 5G). The rarefaction curves for all groups reached a plateau, demonstrating that the sequence data sufficiently capture the taxa diversity within the samples analysed in this section, thereby confirming the sequence depth for subsequent diversity analysis (Fig 5A).

Alpha diversity analysis showed that the Chao1 richness estimator did not differ significantly across any group comparisons, indicating similar species richness among all groups (Fig 5B). The Simpson indexes revealed a significant difference only between the T0 and 24-hour groups (Fig 5C), suggesting some variation in community evenness or dominance early in incubation. These results complement findings from the Shannon and Evenness indices: Shannon diversity differed significantly between the T0 group and control group at 24 hours but not at 48 hours (Fig 5D), while Evenness showed significant differences at both time points (Fig 5E) (S3 File – Alfa indexes). Taken together, these metrics suggest that the bacterial community diversity and structure remained stable throughout incubation, with only transient shifts in specific diversity components occurring during the early stages.

Beta diversity analysis demonstrated that laboratory conditions with sucrose feeding influenced the bacterial community composition of the control groups compared to the T0 group. Significant differences were detected by Bray-Curtis (Fig 5F) and weighted unifrac metrics (pseudo-F = 1.787, p-value = 0.05), while unweighted unifrac did not reveal significant differences among groups (pseudo-F = 1.268, p-value = 0.172). Further, SIMPER analysis attributed more than half (53.12%) of the observed community differences between T0 and control groups at 24 and 48 hours to five ASVs, notably *Lactobacillus* (19.11%), *Bombella* (12.92%), *Leuconostoc* (10.34%), *Lactobacillus sp. Mbhoto3* (5.92%), and *Carnimonas* (4.82%) (S4 File – SIMPER analysis). Differential abundance analysis (ANCOM) (S5 File – ANCOM analysis) identified the family *Acetobacteraceae* (w = 39) as the main contributor to these differences.

## 4. Discussion

The effects of neonicotinoid insecticides on bees have been studied extensively, as scientists continue to report mass mortality of these insects in several parts of the world, as well as neurological, immunological, metabolic, and behavioral damage [60–63]. To the best of our knowledge, this is the first study to assess the effects of sublethal concentrations of imidacloprid on the midgut microbiota of the Brazilian stingless bee, *M. scutellaris*. The bacterial community was also compared between the control group (fed with a sucrose solution under laboratory conditions) and the T0 group (validation group of bees not exposed to laboratory conditions and collected directly from the hives).

The core gut microbiota commonly reported as prevalent in social bees (honeybees, bumblebees, and stingless bees) comprises the phylotypes *Snodgrassella*, *Gilliamella*, *Bombilactobacillus Firm-4*, *Lactobacillus Firm-5,* and *Bifidobacterium* [64]. However, these taxa were not dominant in the gut microbiota of *M. scutellaris*. Consistent with this, Sarton-Lohéac et al. [65] found that the core microbiota of Brazilian stingless bees included families such as *Acetobacteraceae*, *Bifidobacteriaceae*, *Enterobacteriaceae*, *Lactobacillaceae*, *Neisseriaceae*, *Orbaceaceae*, *Prevotellaceae*, *Streptococcaceae,* and *Veillonellaceae*. Similarly, Haag et al. [66] identified *Lactobacillaceae*, *Bifidobacteriaceae,* and *Acetobacteraceae* as core bacterial taxa in *Melipona quadrifasciata*. These findings suggest that the core microbiota composition within the *Melipona* genus shows notable differences compared to other social bees.

Furthermore, both Sarton-Lohéac et al. [65] and Haag et al. [66] highlight that the core microbiota of social bees may be more variable than previously thought. Specifically, the genus *Melipona* appears to lack two core members typically found in social bees – *Snodgrassella* and *Gilliamella –* as also reported by Cerqueira et al. [67]. In agreement with this, our study revealed that *Lactobacillus*, *Bombella*, and *Leuconostoc* were the most prevalent phylotypes. These observations support the hypothesis that the core microbiota of social bees could be variable. Moreover, future research comparing microbiota profiles across multiple *Melipona* species will be essential to clarify genus-specific core microbiota patterns and their functional implications.

Our results show no changes in the gut microbiota of *M. scutellaris* after acute, sublethal exposure to imidacloprid. Similarly, Raymann et al. [68] observed no significant changes in the gut bacterial microbiota composition of honeybees after three days of exposure to sublethal doses of imidacloprid. Almasri et al. [69] also reported that newly emerged honeybees exposed for five days to low doses of imidacloprid, glyphosate, and difeconazole, or a ternary mixture showed no alterations in the core bacterial communities during early gut colonization. Likewise, Balbuena et al. [70] found that Africanized honeybees exposed to sublethal doses of imidacloprid for seven days did not have their microbiota affected. Similarly, exposure to other pesticides, such as spinetoram and glyphosate, for short durations (48 hours) did not affect the microbiota of B. *terrestris* [71].

Importantly, the duration and timing of pesticide exposure appear to play a key role in shaping microbiome responses. In stingless bees, the literature reports that bacterial abundance is altered in *P. helleri* adults following larval exposure to spinosad and glyphosate; additionally, copper sulfate has been shown to reduce bacterial diversity [41]. Botina et al. [42] also reported that spinosad increases the abundance of *Gilliamella* spp. in *P. helleri*. It is important to note that in these studies, *P. helleri* larvae were exposed to pesticides, and the microbiome was evaluated in three-day-old adult bees. Considering that the developmental cycle of *P. helleri* is approximately 46 days [72], this constitutes chronic exposure, which contrasts with our experimental design involving acute exposure (24- and 48-hour) in forager bees. These differences in exposure duration and developmental stage are likely to influence the sensitivity of the gut microbiota to pesticides, reinforcing that microbiome responses may vary depending on species, pesticide type, life stage, and whether exposure is chronic or acute.

While short-term exposure to pesticides may not affect the gut microbiota, chronic exposure over a prolonged period could potentially have negative impacts on gut microbiota. Studies have shown that neonicotinoids can alter gut microbiota within varying timeframes, such as seven days [73], 14 days [74], 18 days [75], 28 days [76], and 35 days [77]. The effects of pesticides on microbiome disruption remain unclear, but current evidence suggests that neonicotinoids may act indirectly via host immune dysregulation and at non-toxic concentrations to bee physiology [78]. Although the bee microbiota has the potential to contribute to xenobiotic detoxification [3], little is known about the conditions under which pesticides could compromise the gut microbiota. Thus, the concentration and/or exposure time reported in the present study may not be sufficient to induce disturbances in the midgut microbiota of *M. scutellaris*. However, this does not mean that imidacloprid does not affect bee health, as several reports using other approaches have demonstrated effects such as cellular damage [79], alterations in protein profiles [80], changes in locomotion activity [81], impairment of memory and brain metabolism [82], and chronic and acute mortality in honeybees [83–86], among others.

Some studies have shown that the type of food available to bees can also modify their microbiota composition. For instance, Sarton-Lohéac et al. [65] linked variation in stingless bee core bacteria to factors such as season, bee age, and development, while Haag et al. [66] reported microbiota shifts in *M. quadrifasciata* following a pollen change from *Myrtaceae* (e.g., *Myrciaria*, *Eugenia*) to *Eucalyptus*. Similarly, gut evaluation of nurses, foragers, and winter honeybees showed that pollen consumption by nurses and winter bees increased gut weight and bacterial communities [87].

The diet-related microbiota changes support our findings of distinct differences in gut bacterial diversity and richness between the control groups fed on sucrose solution and the T0 group (wild-collected forager bees). The artificial sucrose diet (50% w:v) notably altered microbiome composition, primarily through an increased abundance of *Acetobacteraceae*. Bees cannot digest sucrose and convert it to monosaccharides using enzymes secreted by hypopharyngeal glands [88]. *Acetobacteraceae* thrive in a sugar-rich environment by oxidative fermenting oligosaccharides like sucrose, establishing a symbiotic relationship with insects for energy gain [89–91]. Consistent with our results, studies have shown that the type of food offered to bees can influence microbial composition. Powell et al. [92] reported higher bacterial abundance in pollen-fed worker bees compared to those on artificial diet, while Wang et al. [93] found that honey or high-fructose diets increased hindgut microbial diversity relative to those fed sucrose. Moreover, microbiota-depleted bees exhibited higher mortality unless supplemented with natural diets such as honey and pollen [94]. In line with this, *M. scutellaris* foragers collected to compose the T0 group showed greater midgut microbiota diversity and abundance than sucrose-feed controls, with elevated *Acetobacteraceae* reinforcing the link between diet and microbial composition. Together, these findings highlight how artificial diets can reshape the gut microbiota, potentially impacting bee health and resilience.

In this study, we found that the microbiota diversity of *M. scutellaris* changed after 24 hours under experimental conditions, and the abundance changed at both the 24 and 48-hour time points. We hypothesize that these changes resulted from transferring bees to laboratory conditions and feeding them a sucrose solution. Thus, we cannot attribute any of the observed changes in the microbiome to exposure to sublethal concentrations of imidacloprid, as similar changes were observed in the control groups with the same diet and conditions. Our findings do not necessarily exclude the possibility that imidacloprid affects the microbiota, especially in foraging bees. This is because the microbiota of foraging bees differed from that of bees fed sucrose solution, and the significant change in the microbiota due to feeding may overshadow the effect of the insecticide. Although several studies report microbiota disruption after lethal and sublethal insecticide exposure [35–39,76,95,96], many others fail to confirm such effects, and not all studies compare pesticide-treated bees to controls receiving sucrose diets. Therefore, microbiota results require cautious interpretation and should be complemented by additional approaches to ensure assessing pesticide toxicity. Furthermore, further research is needed to understand whether *M. scutellaris* harbors a transient rather than a core microbiota, and to elucidate how food supply under laboratory conditions may affect microbiota studies.

A key limitation of the present study is that microbiota evaluation was performed under short-term pesticide exposure. Risk assessments for bees have traditionally focused on acute toxicity and mortality endpoints, but over the past decade, this approach has been increasingly questioned, with growing recognition of the need to include non-*Apis* species and alternative indicators of harm [47,97–100]. Our findings reinforce the notion that, when investigating the gut microbiota, current short-term diagnostic protocols may be insufficient to detect more subtle or delayed effects of pesticide exposure. We hypothesize that rapid assessments may fail to capture the full extent of microbiota responses, particularly under conditions that do not simulate a realistic chronic exposure scenario.

Currently, two main approaches are used to detect microbiome alterations: comparing exposed versus unexposed groups and comparing microbiota-depleted with non-depleted bee groups [101]. In both cases, analyses typically focus on microbial diversity [102]. Our study follows the same general approach and, likewise, found no detectable changes in gut microbiota following short-term pesticide exposure. However, integrating microbiome endpoints into environmental risk assessments remains challenging [102], particularly due to the lack of a model organism, the absence of standardized and validated OECD protocols, and the need to define a core microbiota [103]. Recently, Rosa-Fontana et al. [103] reviewed these challenges and proposed practical steps for incorporating microbiome data into regulatory frameworks. Their suggestions include using OECD guideline 245 for chronic exposure tests, implementing acclimation periods, and adopting a reference bee for microbiota studies. Such advancements are essential for bridging the gap between microbiome science and ecotoxicological regulation. We believe that, even though no significant differences were observed in the bacterial communities between exposed and control groups in our study, our findings reinforce the relevance of this proposal and support the adoption of chronic exposure frameworks in future microbiome studies.

## Supporting information

S1 File PCoA analysis. PCoA plots based on Weighted Unifrac distances and Shannon Entropy of *Melipona scutellaris* microbiota at different treatments and times of exposure.
(PDF)

S2 File Alpha indexes. Alpha diversity indexes (Chao1, Simpson, Shannon, and Evenness) for each sample after 24 and 48 hours of exposure to sublethal imidacloprid concentrations (LC_50/100_ and LC_50/1000_).
(XLSX)

S3 File Alpha indexes. Alpha diversity indexes (Chao1, Simpson, Shannon, and Evenness) for each sample of T0 and control groups after 24 and 48 hours of exposure.
(XLSX)

S4 File SIMPER analysis. Descriptive statistics to determine the specific ASVs to the observed dissimilarity between the control groups at 24 and 48 h × the T0 group.
(XLSX)

S5 File ANCOM analysis. Descriptive statistics to determine differentially abundant taxa between the control groups at 24 and 48 h × the T0 group.
(XLSX)
