# Supplementary material for: Assessment of the midgut microbiota in foragers of the stingless bee Melipona scutellaris following short-term sublethal exposure to imidacloprid
Source: PLoS One. 2025 Dec 29;20(12):e0339982. doi: 10.1371/journal.pone.0339982 (PMC12747329; doi:10.1371/journal.pone.0339982)
Supplement: S1 File — PCoA plots based on Weighted Unifrac distances and Shannon Entropy of Melipona scutellaris microbiota at different treatments and times of exposure. (PDF) [file pone.0339982.s001.pdf]

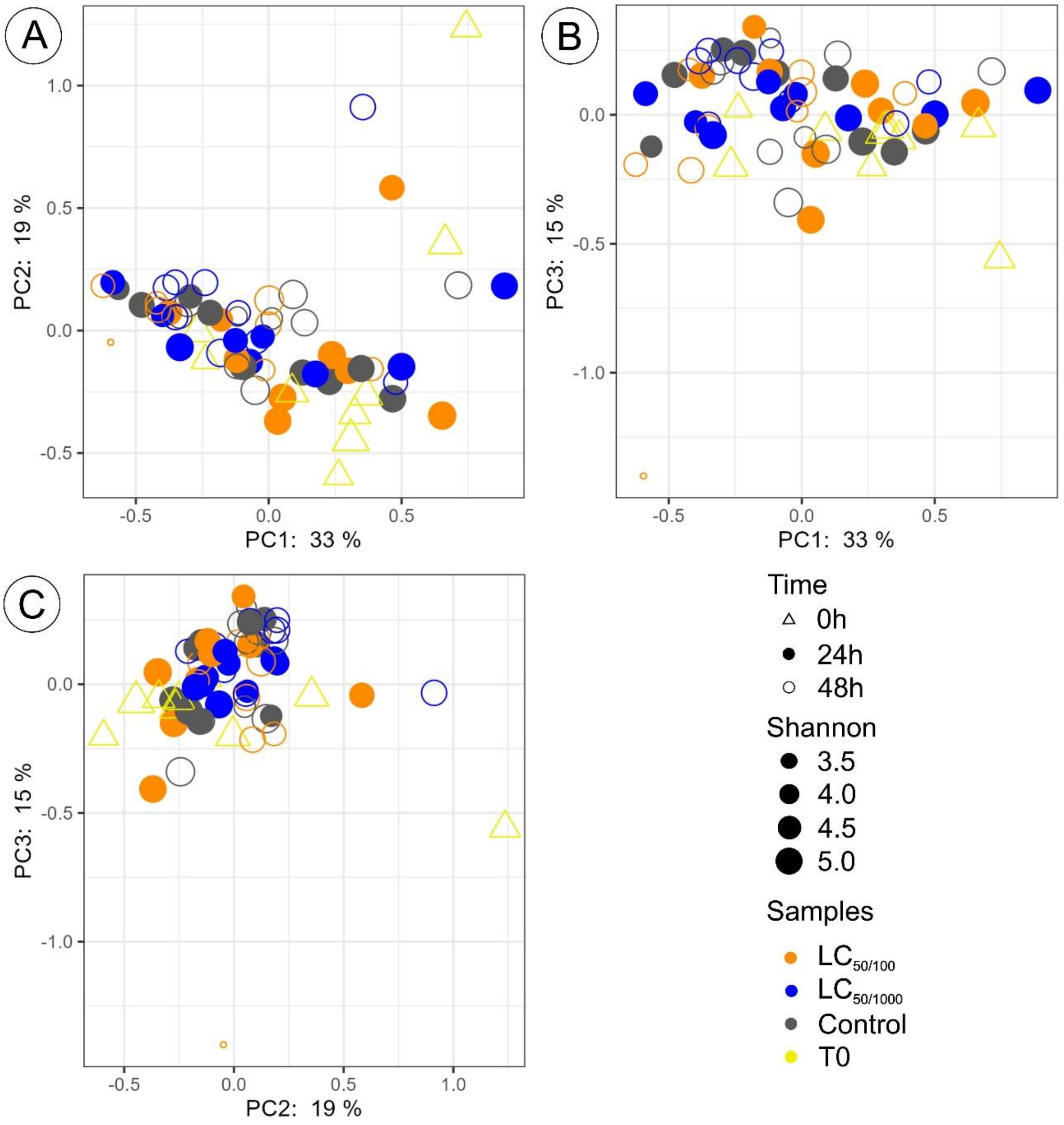

S1. PCoA analysis. PCoA plots based on Weighted Unifrac distances and Shannon Entropy of *Melipona scutellaris* microbiota at different treatments and times of exposure: bees exposed to 0.021 ng a.i./µL ($CL_{50/100}$) in sucrose solution, 0.0021 ng a.i./µL ($CL_{50/1000}$) in sucrose solution, only sucrose solution (CONTROL), or T0 (bees not exposed to any laboratory conditions). (A) PC1 versus PC2; (B) PC1 versus PC3; (C) PC2 versus PC3. The number in the axis titles represents the percentage of variation explained by each axis. PC – Principal Coordinate; PCoA – Principal Coordinate Analysis.
